# Safety and Efficacy of Ketogenic Diet in the Management of Multiple Sclerosis: A Systematic Review

**DOI:** 10.7759/cureus.89965

**Published:** 2025-08-13

**Authors:** Shradha P Kakde, Ahsan Qadeer, Mazhar Khalil, Archana Dhami, Zarwa Rashid, Danish Zahir, Syed Ali Ahsan, Talha A Dhillon, Muhammad Uzair Siddique, Michelle C Uzokwe

**Affiliations:** 1 Internal Medicine, Mahatma Gandhi Mission Institute of Health Sciences, Aurangabad, IND; 2 Internal Medicine, King Edward Medical University, Lahore, PAK; 3 Medicine, Hayatabad Medical Complex, Peshawar, PAK; 4 Family Medicine, Avalon University School of Medicine, Willemstad, CUW; 5 Medicine, King Edward Medical University, Lahore, PAK; 6 School of Medicine, Columbus Central University, Ladyville, BLZ

**Keywords:** beta-hydroxybutyrate, expanded disability status scale, fatigue, inflammation, ketogenic diet, metabolic intervention, microbiome, multiple sclerosis, neuroinflammation, quality of life

## Abstract

This systematic review examines the safety and efficacy of ketogenic diets (KD) in multiple sclerosis (MS) management. Following Preferred Reporting Items for Systematic reviews and Meta-Analyses (PRISMA) guidelines, we systematically searched five major databases through April 2025, identifying six relevant studies published between 2017 and 2024. These studies, predominantly focusing on relapsing-remitting MS, demonstrate consistent benefits of ketogenic interventions across multiple domains. Findings suggest KD normalizes gut microbiome composition, reduces pro-inflammatory markers (ALOX5, COX1, COX2), improves body composition with favorable changes in adipokines (decreased leptin, increased adiponectin), and enhances clinical outcomes, including fatigue, depression, sleep quality, and quality of life measures. Objective neurological improvements were observed in Expanded Disability Status Scale (EDSS) scores, walking ability, and manual dexterity. Adherence rates were notably high, with benefits partially persisting after intervention completion. The metabolic shift induced by ketosis may provide alternative neuronal energy substrates, reduce oxidative stress, and attenuate neuroinflammation. Despite limitations including small sample sizes and study heterogeneity, these preliminary findings suggest KDs represent a promising adjunctive approach in MS management, addressing both inflammatory and neurodegenerative components of the disease. Larger, controlled trials with longer follow-up periods are warranted to establish long-term safety and efficacy profiles.

## Introduction and background

Multiple sclerosis (MS) is a chronic, immune-mediated neurological disorder characterized by inflammation, demyelination, and neurodegeneration within the central nervous system (CNS). It predominantly affects young adults, particularly women, and is a leading cause of non-traumatic neurological disability in this age group [1]. The disease course of MS is highly variable and is categorized into several clinical subtypes, most commonly relapsing-remitting MS (RRMS), which may eventually progress to secondary progressive MS (SPMS), and primary progressive MS (PPMS), where disease progression is continuous from the outset [2]. Despite the availability of disease-modifying therapies (DMTs) that reduce relapse rates and delay disease progression, the burden of MS remains substantial due to its unpredictable nature, incomplete therapeutic responses, and persistent symptoms, including fatigue, cognitive impairment, and gait abnormalities [3]. MS pathogenesis is driven by a complex interplay between genetic susceptibility and environmental triggers that initiate an aberrant immune response targeting myelin and oligodendrocytes. While the exact etiology remains elusive, recent evidence suggests that metabolic dysregulation and mitochondrial dysfunction play a central role in the neurodegenerative aspects of MS. As such, nutritional and metabolic interventions have gained interest as adjunctive strategies to mitigate disease progression and improve symptom burden [4].

The ketogenic diet (KD), a high-fat, low-carbohydrate, and adequate-protein dietary regimen, has long been used in the management of refractory epilepsy and is now being investigated in a variety of neurological and neurodegenerative conditions [5]. By inducing a state of physiological ketosis, wherein ketone bodies such as β-hydroxybutyrate (BHB) and acetoacetate become the primary energy substrates, KD aims to bypass impaired glucose metabolism and provide alternative fuel sources for neurons. Moreover, ketone bodies exhibit multiple neuroprotective properties, including attenuation of oxidative stress, modulation of mitochondrial function, suppression of pro-inflammatory pathways, and enhancement of synaptic plasticity [6].

In the context of MS, KD is hypothesized to exert beneficial effects through several mechanistic pathways. Animal models of MS, such as experimental autoimmune encephalomyelitis (EAE), have demonstrated that KD can reduce demyelination, inhibit microglial activation, and promote remyelination. These effects are mediated in part through reduction in reactive oxygen species (ROS), inhibition of the NLRP3 inflammasome, and increased production of brain-derived neurotrophic factor (BDNF) [7]. Clinically, ketogenic interventions may offer symptomatic improvements in fatigue, cognition, and mood, which are among the most disabling yet poorly addressed features of MS.

Despite its promising theoretical underpinnings, the clinical evidence supporting the use of KD in MS remains nascent and heterogeneous. Preliminary studies, including small-scale trials and observational cohorts, have reported variable outcomes with respect to safety, adherence, and efficacy. While some investigations highlight improvements in quality of life, Expanded Disability Status Scale (EDSS) scores, and inflammatory markers, others report no significant differences or note challenges with dietary adherence [6,8,9]. Furthermore, the restrictive nature of KD raises important concerns about its long-term sustainability, nutritional adequacy, and potential adverse effects, such as hyperlipidemia, gastrointestinal discomfort, and nephrolithiasis. These concerns necessitate a careful appraisal of both the therapeutic potential and risks associated with ketogenic dietary interventions in MS patients.

In addition, the psychosocial and behavioral dimensions of implementing KD in a chronic, disabling disease like MS cannot be overlooked. Given the high rates of fatigue, depression, and cognitive impairment in MS patients, adherence to a rigid dietary protocol may be particularly challenging [10]. The role of supportive interventions, such as dietitian-led counseling and digital tracking tools, has not been systematically evaluated in this population. Furthermore, the heterogeneity in dietary protocols classified as “ketogenic”, ranging from classical 4:1 fat-to-carbohydrate ratios to modified Atkins and low glycemic index therapies, complicates the interpretation and comparison of existing studies.

To date, there has been no comprehensive synthesis of the literature that critically examines both the efficacy and safety of KDs in the management of MS across its various subtypes and clinical stages. A systematic evaluation of current evidence is necessary to determine whether KD constitutes a viable adjunctive therapy in MS and to identify the limitations, risks, and gaps in existing research. Such an analysis is particularly timely in an era of increasing patient-driven interest in complementary and lifestyle-based interventions alongside conventional pharmacotherapy.

## Review

Materials and methods

This systematic review was conducted in accordance with the Preferred Reporting Items for Systematic Reviews and Meta-Analyses (PRISMA) 2020 guidelines to ensure transparency and rigor in study identification, screening, eligibility assessment, and data extraction.

Search Strategy

A comprehensive literature search was performed across five major electronic databases: PubMed, Scopus, Web of Science, EMBASE, and Hinari from database inception through April 2025. The search strategy employed a combination of Medical Subject Headings (MeSH) and free-text keywords to capture relevant studies. Search terms included “ketogenic diet,” “ketosis,” “low carbohydrate high fat diet,” “LCHF,” “multiple sclerosis,” “relapsing-remitting multiple sclerosis,” “RRMS,” “autoimmune demyelination,” “clinical trials,” “safety,” and “efficacy.” Boolean operators such as “AND” and “OR” were used to combine the terms appropriately. The search strategy was tailored for each database. To enhance the comprehensiveness of the review, reference lists of all included studies and related systematic reviews were manually screened to identify any relevant articles that may have been missed in the initial database search.

Eligibility Criteria

We included peer-reviewed studies that investigated the safety and/or efficacy of the KD in individuals diagnosed with MS, irrespective of the MS subtype. Eligible study designs included randomized controlled trials (RCTs), quasi-experimental studies, cohort studies, case-control studies, and prospective or retrospective observational studies. Studies were included if they reported any relevant clinical, biochemical, or patient-reported outcomes such as disability progression (e.g., EDSS score), relapse rate, fatigue, cognition, quality of life, inflammatory markers, neuroimaging findings, adverse events, or adherence rates. We excluded animal studies, case reports, reviews, editorials, conference abstracts, and studies not published in English. In addition, studies that used KD for other neurological conditions or general lifestyle interventions without a clear delineation of MS outcomes were excluded. Only studies involving adult patients (≥18 years of age) with a confirmed diagnosis of MS were considered.

Study Selection

All retrieved records were imported into the Rayyan software for duplicate removal. Following de-duplication, two independent reviewers screened the titles and abstracts of all remaining studies for potential eligibility. The full texts of studies deemed relevant or potentially relevant were then assessed independently by the same reviewers. Discrepancies in selection were resolved through consensus discussion and, if required, adjudication by a third reviewer. A PRISMA flow diagram was used to document the selection process, including reasons for exclusion at each stage.

Data Extraction

Data extraction was conducted independently by two reviewers using a predefined and piloted data extraction form. The following variables were extracted: first author, year of publication, country of study, study design, sample size, MS subtype (e.g., RRMS, SPMS), outcome measures assessed (e.g., relapse rate, EDSS score, MRI findings, fatigue severity, inflammatory biomarkers, adherence, and side effects), and key results. Where possible, both baseline and post-intervention values were extracted. Discrepancies in data extraction were resolved by consensus and, if necessary, by referral to a third reviewer.

Data Synthesis

Due to the anticipated heterogeneity in study designs, intervention protocols (e.g., classical KD vs. modified Atkins diet), outcome definitions, and duration of follow-up, a quantitative meta-analysis was not planned. Instead, a qualitative narrative synthesis was undertaken. Extracted outcomes were summarized in tabular format and described according to clinical efficacy (e.g., relapse rate, disability progression), patient-reported symptoms (e.g., fatigue, cognition), safety and tolerability (e.g., dropout rates, adverse events), and biochemical or imaging markers (e.g., inflammatory cytokines, MRI lesions). Key trends, consistencies, and discrepancies among the studies were highlighted. Where possible, subgroup observations (e.g., effect based on MS subtype or diet duration) were noted. Limitations of individual studies and gaps in the literature were also documented as part of the synthesis.

Results

Study Selection

**Figure 1:**
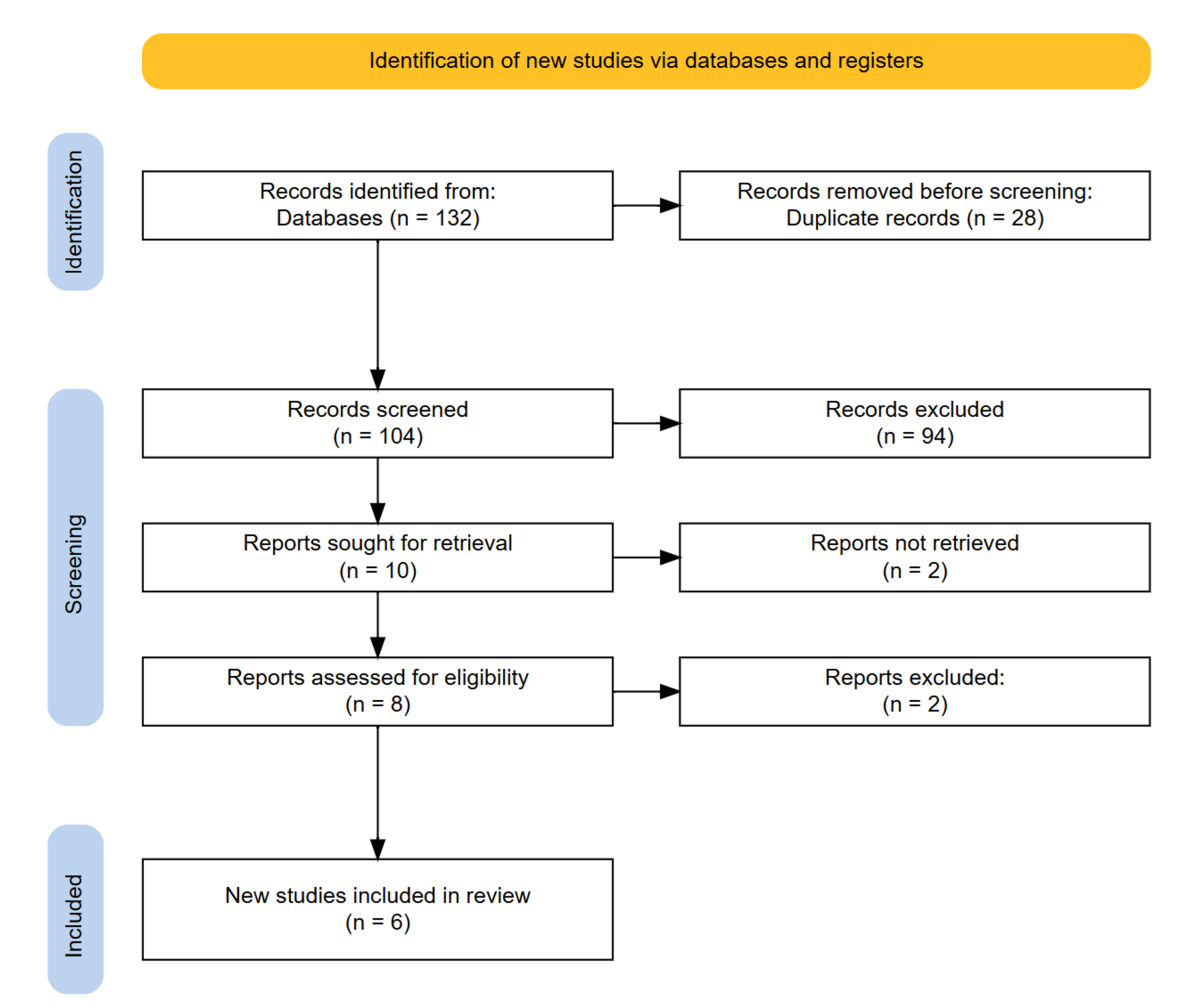
PRISMA diagram illustrating the study selection process.

The initial search across PubMed, Scopus, Web of Science, Hinari, and EMBASE databases yielded 132 records. After removal of 28 duplicates, a total of 104 unique studies remained for title and abstract screening. During this phase, 94 studies were excluded for reasons including irrelevance to the research question, use of non-ketogenic interventions, focus on other neurological conditions, or inadequate outcome reporting. The remaining 10 studies were subjected to full-text review. After thorough assessment, four studies were excluded due to reasons such as insufficient clinical data on MS outcomes, unclear KD protocols, case report design, or lack of safety and efficacy endpoints. Ultimately, six studies met all inclusion criteria and were included in the final qualitative synthesis. The complete study selection process is illustrated in the PRISMA 2020 flow diagram (Figure 1).

Study Characteristics

The manuscript reviewed six studies investigating KD interventions for MS patients published between 2017 and 2024. The studies represent a diverse set of methodological approaches, including comparative intervention studies, randomized clinical trials, pilot studies with quasi-experimental designs, prospective intention-to-treat studies, exploratory clinical studies, and prospective follow-up studies. Sample sizes ranged from 21 to 65 subjects, with most studies focused on relapsing-remitting MS patients. Study durations typically spanned six months, with one including a three-month post-intervention follow-up [11]. The studies measured a comprehensive range of outcomes, including microbiome composition, inflammatory markers, anthropometric measurements, disability progression (EDSS scores), quality of life metrics, fatigue levels, depression scores, walking and dexterity tests, sleep quality, and adherence rates. Key findings consistently demonstrated several benefits of KD: normalization of microbiome composition, reduction in inflammatory markers, improvements in body composition (decreased fat mass, increased lean mass), significant reductions in fatigue and depression symptoms, enhanced quality of life scores, improved neurological function, and better sleep quality. Adherence rates were generally high during interventions, with some participants maintaining dietary changes after study completion, suggesting KD can be a sustainable intervention for MS patients with potentially lasting benefits (Table 1).

**Table 1:** Summary of the main findings of included studies. MS: Multiple Sclerosis, FISH: Fluorescence In Situ Hybridization, rRNA: ribosomal RNA, EREC: Eubacterium rectale-Clostridium coccoides group, Bac303: Bacteroides, Fprau: Faecalibacterium prausnitzii, CR: Caloric Restriction, AKD: Atkins Ketogenic Diet, MSQOL-54: Multiple Sclerosis Quality of Life-54, ALOX5: Arachidonate 5-Lipoxygenase, ALOX15: Arachidonate 15-Lipoxygenase, COX1: Cyclooxygenase-1, COX2: Cyclooxygenase-2, BMI: Body Mass Index, VAS: Visual Analog Scale, BHB: Beta-Hydroxybutyrate, PON1: Paraoxonase 1, EDSS: Expanded Disability Status Scale, 6MW: Six-Minute Walk Test, MS QoL: Multiple Sclerosis Quality of Life, PSQI: Pittsburgh Sleep Quality Index, ESS: Epworth Sleepiness Scale, DASS-21: Depression Anxiety Stress Scales-21, EDS: Excessive Daytime Sleepiness, KD: Ketogenic Diet, MSFC: Multiple Sclerosis Functional Composite, SDMT: Symbol Digit Modalities Test, LCVA: Low Contrast Visual Acuity

Author	Year	Study design	Sample size	Outcomes studied	Main findings	Conclusions
Swidsinski et al. [12]	2017	Comparative study with intervention component	25 MS patients and 14 healthy controls; 10 MS patients selected for KD intervention	Composition of colonic microbiota using fluorescence in situ hybridization (FISH) with 162 rRNA bacterial probes	No MS-specific microbiome pattern was found. Total bacterial concentrations and diversity were reduced in MS patients compared to healthy controls (P < 0.001). Bacterial groups detected with EREC (mainly Roseburia), Bac303 (Bacteroides), and Fprau (Faecalibacterium prausnitzii) probes were diminished the most. KD effects were biphasic: short-term reduction in bacterial concentrations and diversity, followed by recovery exceeding baseline values after 23-24 weeks.	Colonic biofermentative function is markedly impaired in MS patients. The KD normalized concentrations of the colonic microbiome after 6 months. Microbiome alterations in MS are not inherent and can be corrected through dietary intervention
Bock et al. [13]	2018	Three-armed, parallel grouped, single centered, controlled and randomized clinical pilot trial	60 adults initially recruited; data analyzed for 24 patients (8 controls, 5 on CR and 11 on AKD)	MS quality of life-54 (MSQOL-54) index, gene expression of eicosanoid biosynthesis enzymes (ALOX5, ALOX15, COX1, COX2) in peripheral leukocytes	Significant reduction in pro-inflammatory ALOX5 expression in the KD groups compared to control (p < 0.05). Significant decreases in COX1 (p < 0.001) and COX2 (p < 0.05) expression within the KD groups. Inverse correlation between ALOX5/COX1 expression and MSQOL-54 index. Significant increase in ketone bodies and decrease in BMI in KD groups.	KDs can reduce the expression of enzymes involved in the biosynthesis of pro-inflammatory eicosanoids. Pharmacological interference with eicosanoid biosynthesis might constitute a supplementary therapeutic strategy for MS treatment.
Benlloch et al. [14]	2019	Pilot study with quasi-experimental design (prospective, mixed)	27 MS patients	Anthropometric measurements, satiety/hunger perception (VAS scale), beta-hydroxybutyrate (BHB) levels, paraoxonase 1 (PON1) as oxidation marker, ghrelin levels	Significant increase in satiety perception at lunch and dinner. Significant increase in BHB in blood. Significant decrease in hunger perception at lunch and dinner. Significant increase in lean mass. Significant decrease in fat mass. Significant increase in PON1 levels. No significant change in ghrelin levels.	A Mediterranean isocaloric KD increases lean mass and decreases inflammation and oxidation, possibly as a consequence of increased satiety and decreased hunger in MS patients. This suggests KDs may offer metabolic benefits for MS patients through multiple mechanisms, including their satiating effect.
Brenton et al. [9]	2022	Phase II prospective, intention-to-treat intervention study	65 subjects enrolled (64 analyzed, 53 completed with adherence)	Adherence to KD, body composition, fatigue & depression scores, quality of life, neurological disability (EDSS), walking tests (6MW), dexterity (nine-hole peg test), blood markers (including leptin and adiponectin)	83% adherence rate for the 6-month study period. Significant reductions in fat mass. ~50% decline in self-reported fatigue and depression. Improved MS QoL physical health (67±16 vs 79±12, p<0.001) and mental health (71±17 vs 82±11, p<0.001). Improved EDSS scores (2.3±0.9 vs. 1.9±1.1, p<0.001). Improved 6-minute walk (1631±302 vs. 1733±330 ft, p<0.001). Improved Nine-Hole Peg Test (21.5±3.6 vs. 20.3±3.7 s, p<0.001). Reduced serum leptin (25.5±15.7 vs. 14.0±11.7 ng/mL, p<0.001). Increased adiponectin (11.4±7.8 vs. 13.5±8.4 μg/mL, p=0.002).	KDs are safe and tolerable over a 6-month study period and yield improvements in body composition, fatigue, depression, quality of life, neurological disability, and adipose-related inflammation in persons living with relapsing MS. The findings support the rationale for a large-scale randomized controlled trial of KD as a complementary treatment for MS.
Merlino et al. [15]	2023	Exploratory clinical study (open-label, single-arm, prospective)	21 patients with relapsing-remitting MS (initially enrolled), with 13 completing the study	Sleep quality (PSQI), excessive daytime sleepiness (ESS), psychological status (DASS-21), quality of life (MSQoL-54), anthropometric measures (BMI, fat mass, fat-free mass)	Global PSQI score decreased (T0: 7.7 ± 3.1 vs. T1: 4.4 ± 3.1, p = 0.002). ESS scores decreased (T0: 7.5 ± 3.9 vs. T1: 4.9 ± 3.2, p = 0.001). Poor sleep prevalence reduced from 66.7% to 23.8%. EDS prevalence reduced from 38.1% to 9.5%. Improved psychological status (depression, anxiety, stress). Improved QoL metrics, especially the mental health composite. Reduced BMI and body composition measures.	KD may improve sleep complaints (poor sleep quality and daytime sleepiness) in non-disabled or minimally disabled patients with relapsing-remitting MS. KD positively impacts psychological status and QoL in MS patients, mainly through improving sleep quality. Further controlled studies with larger sample sizes are needed to confirm these preliminary results
Wetmore et al. [11]	2024	Prospective follow-up study (3-month post-intervention evaluation following a 6-month KD intervention trial)	65 subjects with relapsing MS enrolled; 52 subjects (81%) completed the 3-month post-intervention follow-up	Diet adherence post-intervention, patient perceptions of KD, anthropometric measures (BMI, waist circumference), patient-reported outcomes (fatigue, depression, quality of life), clinical outcomes (EDSS, MSFC, SDMT, 6MW, LCVA), laboratory values (vitamin D, lipid profiles, adipo-cytokines)	21% maintained strict KD post-intervention; 37% followed a liberalized KD. Greater BMI reduction and fatigue improvement were associated with continued KD adherence. Most intervention benefits persisted 3 months post-trial but were attenuated. Dietary patterns shifted toward more protein and polyunsaturated fats and fewer carbohydrates/added sugars regardless of post-study diet. 47% reported improvement in MS-related symptoms during KD. 23% reduced or stopped medications during the KD trial. Most reported KD benefits were weight loss (62.5%) and increased energy/focus (28.5%).	KDs are sustainable short-term outside clinical trial settings for MS patients. The majority of clinical and patient-reported benefits obtained during the KD intervention were sustained at 3 months post-intervention, though somewhat attenuated. A 6-month KD intervention induced persistent changes to dietary habits with greater allocation toward protein and polyunsaturated fats and less toward carbohydrates and added sugars.

Quality Assessment

Study quality was evaluated using the Newcastle-Ottawa Scale (NOS) for non-randomized studies, assessing three domains: selection of study groups, comparability of groups, and ascertainment of outcomes. The NOS tool assesses the quality of studies based on three domains: selection of study groups, comparability of groups, and ascertainment of outcomes. Two independent reviewers performed quality assessments, with disagreements resolved through consensus. Overall study quality was moderate to high, with scores ranging from 6 to 8 out of 9 possible points. Most studies demonstrated adequate participant selection and outcome assessment, though comparability was limited by the absence of control groups in several studies. The small sample sizes and short follow-up periods were consistent limitations across studies, potentially affecting the robustness of findings (Table 2).

**Table 2:** Quality assessment of included studies using the Newcastle-Ottawa Scale (NOS).

Author	Selection	Comparability	Outcome	Total NOS Score	Quality Rating
Swidsinski et al. [12]	⭐⭐⭐	⭐⭐	⭐⭐	7/9	Good
Bock et al. [13]	⭐⭐⭐	⭐⭐	⭐⭐⭐	8/9	Good
Benlloch et al. [14]	⭐⭐	⭐	⭐⭐⭐	6/9	Satisfactory
Brenton et al. [9]	⭐⭐⭐	⭐⭐	⭐⭐⭐	8/9	Good
Merlino et al. [15]	⭐⭐	⭐	⭐⭐⭐	6/9	Satisfactory
Wetmore et al. [11]	⭐⭐⭐	⭐⭐	⭐⭐⭐	8/9	Good

Discussion

The findings from the six studies included in this systematic review provide preliminary evidence supporting the safety and potential efficacy of KDs as an adjunctive intervention for MS management. Several key themes emerge from the collective data that warrant further exploration.

First, the consistent improvements in inflammatory markers observed across multiple studies suggest that KDs may modulate the immune dysregulation characteristic of MS. Bock et al. demonstrated significant reductions in pro-inflammatory eicosanoid biosynthesis enzymes (ALOX5, COX1, and COX2) in peripheral leukocytes, which correlated with improved quality of life scores [13]. This aligns with the known anti-inflammatory properties of ketone bodies, particularly β-hydroxybutyrate, which has been shown to inhibit the NLRP3 inflammasome and reduce oxidative stress. The normalization of colonic microbiota composition reported by Swidsinski et al. further supports the notion that KDs may attenuate inflammatory processes through modulation of the gut-brain axis, an increasingly recognized pathway in MS pathogenesis [12]. Second, the improvements in body composition, specifically decreased fat mass and increased lean mass, observed in multiple studies (Benlloch et al., Brenton et al.) are particularly relevant in the context of MS [9,14]. Adipose tissue is not merely a passive energy storage site but an active endocrine organ that secretes pro-inflammatory adipokines. The significant reduction in leptin and increase in adiponectin levels reported by Brenton et al. suggest that KDs may favorably alter the adipokine profile, potentially contributing to reduced systemic inflammation and improved metabolic health in MS patients [9]. This is especially important considering the emerging evidence linking obesity and metabolic dysfunction to MS risk and progression.

Perhaps most compelling are the improvements in clinical and patient-reported outcomes. The significant reductions in fatigue and depression scores, along with enhanced quality of life metrics, address some of the most debilitating yet often undertreated aspects of MS. Fatigue, which affects up to 90% of MS patients, showed approximately 50% improvement in the Brenton et al. study [9]. Similarly, Merlino et al. reported substantial improvements in sleep quality and daytime sleepiness, with the prevalence of poor sleep decreasing from 66.7% to 23.8% [15]. These findings are particularly noteworthy, as fatigue, depression, and sleep disturbances significantly impact daily functioning and quality of life in MS patients, often persisting despite DMTs. The observed improvements in objective measures of neurological function, including EDSS scores, walking distance, and manual dexterity, suggest that KDs may influence not only symptomatic manifestations but potentially the underlying neurodegenerative processes in MS. The mechanism may involve enhanced mitochondrial function and energy metabolism in neurons, provision of alternative fuel substrates (ketones) that bypass impaired glucose metabolism, and possibly neuroprotective effects mediated through increased BDNF production and reduced oxidative stress.

The sustainability of ketogenic interventions, often a concern with restrictive dietary protocols, appears promising based on the high adherence rates (83%) reported by Brenton et al. and the post-intervention dietary patterns observed by Wetmore et al. [9,11]. The finding that 58% of participants maintained either a strict or liberalized KD three months after study completion indicates that such interventions may be feasible in real-world settings, particularly when patients experience tangible benefits. The persistence of many clinical improvements three months post-intervention, albeit somewhat attenuated, further suggests that KDs may induce lasting metabolic adaptations rather than transient changes contingent upon strict dietary adherence. Interestingly, 23% of participants in the Wetmore et al. study reported reducing or discontinuing medications during the KD intervention [11]. While this observation must be interpreted cautiously without controlled comparison, it raises intriguing questions about potential drug-diet interactions and the possibility that KDs might enhance the efficacy of conventional pharmacotherapies or address symptoms refractory to standard treatments.

The metabolic benefits of KDs may be particularly relevant in MS, given the growing recognition of metabolic dysfunction as a contributor to neurodegeneration. By shifting metabolism from glucose dependence to ketone utilization, KDs may bypass impaired glucose metabolism in the CNS, provide more efficient energy substrates for neurons, reduce oxidative stress through enhanced mitochondrial function, and possibly enhance remyelination processes through increased production of lipid substrates.

Limitations and future directions

Despite these promising findings, several limitations must be acknowledged. The small sample sizes (ranging from 21 to 65 participants) limit statistical power and generalizability. Most studies focused on relapsing-remitting MS, leaving uncertainties about efficacy in progressive forms of the disease. The absence of control groups in several studies and the heterogeneity in KD protocols (modified Atkins, Mediterranean ketogenic, etc.) complicate interpretation. Additionally, the relatively short intervention periods (predominantly six months) preclude conclusions about long-term safety and efficacy.

Future research should prioritize larger randomized controlled trials with longer follow-up periods, inclusion of diverse MS phenotypes, standardized ketogenic protocols, and comprehensive outcome measures, including advanced neuroimaging and metabolomics. Mechanistic studies elucidating the molecular pathways mediating KD effects in MS are needed. Practical considerations for implementation, including strategies to enhance adherence and minimize side effects, warrant investigation. Finally, studies examining the interaction between KDs and DMTs could provide valuable insights for integrated treatment approaches.

## Conclusions

This systematic review provides preliminary evidence supporting the safety and potential efficacy of ketogenic diets as complementary interventions in MS management. The consistent improvements observed across inflammatory markers, body composition, neurological function, and patient-reported outcomes suggest that ketogenic metabolic states may favorably modulate multiple pathophysiological aspects of MS. The high adherence rates and persistence of benefits after intervention completion further indicate clinical viability. However, the current evidence base is limited by small sample sizes, methodological heterogeneity, and short follow-up periods. Ketogenic diets appear to offer a multifaceted approach that addresses both the inflammatory and neurodegenerative components of MS pathogenesis, potentially filling an important gap in current therapeutic strategies. Larger randomized controlled trials with standardized protocols and comprehensive outcome measures are urgently needed to definitively establish the role of ketogenic diets in the integrative management of multiple sclerosis.
